# Effect of mineralization of *Erythrina poeppigiana* particles and accelerated carbonation on the properties of fiber cement

**DOI:** 10.1007/s11356-026-37819-4

**Published:** 2026-05-09

**Authors:** Lúcia Maria Joaquim Assane, Ianca Oliveira Borges, Felipe Gomes Batista, Lorran de Sousa Arantes, Murilo Daniel de Mello Innocentini, Ricardo Gabriel de Almeida Mesquita, José Benedito Guimarães Junior, Lourival Marin Mendes

**Affiliations:** 1 Department of Forest Sciences, Federal University of Lavras, Lavras, Brazil; 2 Department of Geosciences and Natural Resource Management, University of Copenhagen, Copenhegen, Denmark; 3 Materials and Environmental Process Optimization Research Group, University of Ribeirão Preto, Ribeirão Preto, Brazil; 4 Department of Architecture and Civil Engineering, University of Bath, Bath, UK; 5 Training Center in Agroforestry Sciences, Federal University of Southern Bahia, Itabuna, Brazil

**Keywords:** Aluminum sulfate, Calcium carbonate, Durability, Accelerated carbonation, Fiber cement composites

## Abstract

Fiber cement is widely used in the construction industry due to its satisfactory physical and mechanical properties. However, the need to enhance these characteristics has driven research focused on the development of new additives and the optimization of manufacturing processes. In this context, the present study aimed to develop and evaluate extruded fiber cement composites reinforced with *Erythrina poeppigiana* particles. The particles were mineralized with 9% aluminum sulfate [Al₂(SO₄)₃] and chemically characterized. The composites were produced using 66.7% Portland cement (CPV-ARI), 28.3% limestone, and 5% particles. After 72 h of curing, the specimens underwent accelerated carbonation for 24 h. Composite analyses were performed after 28 days of curing. The combination of aluminum sulfate treatment and carbonation (STMC) resulted in composites with improved performance, as evidenced by increased flexural strength (MOR), modulus of elasticity (MOE), and toughness, along with a significant reduction in porosity, water absorption, thermal conductivity, and air permeability. These results demonstrate the technical feasibility of the treated composites, indicating their potential for application in construction systems requiring high structural performance and durability.

## Introduction

The growing demand for sustainable practices in the construction industry has driven the development of alternative materials that combine technical performance with reduced environmental impact. In this context, extruded fiber cement emerges as a promising solution, recognized for its favorable mechanical properties, durability, and capacity to incorporate lignocellulosic residues, thereby contributing to the valorization of renewable resources and waste mitigation (Tonoli et al. 2010). Compared to conventional cementitious materials, fiber-reinforced composites exhibit significant improvements in fracture toughness and post-cracking behavior, with reported increases ranging from approximately 2 to over 4 times depending on fiber type and composite design (Shi et al. 2025).

The incorporation of plant fibers into fiber cement is widely recognized as an effective strategy to improve crack bridging, energy absorption, and post-cracking behavior. However, the performance gains are highly dependent on fiber characteristics and interfacial bonding (Naqi et al. 2019; Alencar et al. 2023). Previous studies report that untreated lignocellulosic fibers can lead to reductions of up to 20–40% in long-term mechanical performance due to fiber degradation in alkaline environments (Schiavon and Andrade 2023; Hurtado-Figueroa et al. 2025). This degradation is primarily associated with the dissolution of hemicelluloses and lignin, as well as mineral deposition within the fiber lumen, which compromises fiber integrity and fiber-matrix adhesion over time.

To mitigate these limitations, chemical treatments such as mineralization with aluminum sulfate [Al₂(SO₄)₃] have been proposed to enhance fiber stability. Mineralization promotes the formation of inorganic deposits on the fiber surface, reducing water absorption and limiting alkaline attack. Experimental results indicate that such treatments can reduce water absorption and improve interfacial adhesion, resulting in increases in mechanical strength of up to 4 × compared to untreated fibers (Pantawee et al. 2017; Balčiūnas et al. 2018). Nevertheless, most studies have focused on conventional casting processes, with limited investigation into extruded systems, where fiber orientation and matrix densification play a critical role.

In parallel, accelerated carbonation has gained increasing attention as an advanced curing method for cement-based materials. This process promotes the reaction of CO₂ with hydration products, leading to calcium carbonate (CaCO₃) formation, pore refinement, and densification of the microstructure. Studies have reported significant reductions in total porosity (up to ~ 40%), accompanied by increases in apparent density (≈13%) and substantial improvements in mechanical performance, with strength gains reaching up to ~ 70%, depending on the curing regime and mixture composition (Almeida et al. 2013; Santos et al. 2015; Urrea-Ceferino et al. 2017; Filomeno et al. 2020). In addition to enhancing performance, carbonation enables partial CO₂ sequestration within the cementitious matrix, contributing to the reduction of the overall carbon footprint of these materials (Roijen et al. 2024).

Based on stoichiometric considerations, in cement-based materials, a fraction of the available phases undergo carbonation due to kinetic and microstructural limitations. Experimental and modeling studies report that CO₂ uptake in cementitious systems typically ranges from approximately 0.05 to 0.34 kg CO₂ per kg of cement, corresponding to about 5–30% of the cement mass depending on exposure conditions, porosity, and curing environment (Roijen et al. 2024). At a global scale, carbonation has been shown to offset a significant portion of cement-related emissions, with cumulative CO₂ uptake reaching up to 46% of historical emissions from cement production (Niu et al. 2025).

Despite these advances, the combined effects of fiber mineralization and accelerated carbonation remain poorly understood. While mineralization primarily targets fiber durability and interfacial stability, carbonation acts on matrix densification and pore structure refinement. The potential synergistic interaction between these mechanisms, particularly in extruded composites, where fiber alignment and reduced porosity already influence performance, has not been systematically investigated.

In this context, the use of alternative lignocellulosic sources is also critical. *Erythrina poeppigiana* (Fabaceae, Faboideae) is widely utilized as a shade tree in cocoa and coffee agroforestry systems due to its rapid biomass production, deciduous nature, and ornamental value, as well as its ability to contribute to soil nutrient cycling through nitrogen fixation (Roveda et al. 2021). Despite these traditional uses and its potential ecosystem services, the species is exotic in many regions and has limited commercial application in solid wood products, primarily due to the low natural density and durability of its wood, which significantly restricts its value for conventional timber industries.

From a socioeconomic perspective, cultivating native or multi-purpose species in agroforestry systems can provide broader environmental and economic benefits, as diversified systems tend to deliver more stable ecosystem services and additional sources of income for smallholder farmers. However, *Erythrina poeppigiana* may cause excessive shading if not properly pruned or managed, which in some cases reduces cocoa productivity by competing for light and other resources with cacao plants (Tinoco-Jaramillo et al. 2024). In this context, the use of underutilized biomass in fiber cement composites represents a strategy for valorizing a locally available, low-value biomass while contributing to residue utilization in agroforestry systems.

The present study therefore aimed to evaluate the effects of aluminum sulfate mineralization and accelerated carbonation on the physical, mechanical, permeability, and microstructural properties of extruded fiber-cement composites reinforced with *Erythrina poeppigiana* particles. While previous studies have investigated plant fiber mineralization or accelerated carbonation separately, the novelty of this work lies in the synergistic mechanism of combined application of these approaches in extruded composites reinforced with an underexplored agroforestry species. By linking microstructural, chemical, mechanical, thermal, and permeability performance, this study provides a comprehensive assessment that advances the development of more efficient and environmentally responsible construction materials.

## Materials and methods

### Obtaining the material

For this study, *Erythrina poeppigiana* particles were used as reinforcement in extruded fiber cement composites. Logs measuring 4 m in length were sectioned into billets of approximately 58 cm. These billets were immersed in a heated water tank at 70 °C for 24 h to facilitate the peeling process. Subsequently, veneers with a thickness of 2 mm were produced using a rotary lathe. The veneers were then processed in a hammer mill, resulting in sliver-type particles.

### Characterization of lignocellulosic material

The *Erythrina poeppigiana* particles were classified by particle size through sieving using a set of stacked sieves with openings of 40 mesh (upper) and 60 mesh (lower). For chemical composition analysis, the fraction retained on the 60-mesh sieve was used. The insoluble lignin content was determined according to NBR 7,989 (ABNT 2010a), extractive content following NBR 14,853 (ABNT 2010b), and ash content according to NBR 13,999 (ABNT 2003). The cellulose content was obtained using the method described by Kennedy et al. (1987), whereas holocellulose content was determined following Browning (1963). Hemicellulose content was calculated as the difference between holocellulose and cellulose contents.

### Mineralization of particles

The wood particles were subjected to a mineralization process using an aluminum sulfate [Al₂(SO₄)₃] solution at a concentration of 9% (w/w), the concentration was determined from preliminary tests to find the ideal concentration that would not present particles degradation problems. The mixture was prepared in a planetary mixer, where the particles were combined with the solution and stirred for 3 min. Subsequently, the suspension was allowed to rest for 15 min to promote salt adsorption and initiate surface mineralization of the particles, following a methodology adapted from Pantawee et al. (2017).

### Composite production

The composites were produced on a laboratory scale using the extrusion method. The cementitious matrix consisted of 66.7% Portland cement (CPV-ARI), as specified in NBR 5733 (ABNT 1991). The formulation followed the procedures described by Tonoli et al. (2010) and comprised 5% lignocellulosic material, 28.3% ground agricultural limestone, and 1% of additives, including hydroxypropyl methylcellulose (HPMC) and polyether carboxylate (ADVA), to improve the rheological behavior of the fiber cement mixture (Table 1).

**Table 1:** Experimental plan of fiber cement composites

Treatments	Cement (%)	Limestone (%)	Particles (%)
*Control*	66.7	28.3	5.0
*STM*	66.7	28.3	5.0
*STC*	66.7	28.3	5.0
*STMC*	66.7	28.3	5.0

where control-composite containing natural plant particles; STM-composite with plant particles previously mineralized with Al₂(SO₄)₃; STC-composite subjected to carbonation; and STMC-composite containing mineralized plant particles.

**Fig. 1:**
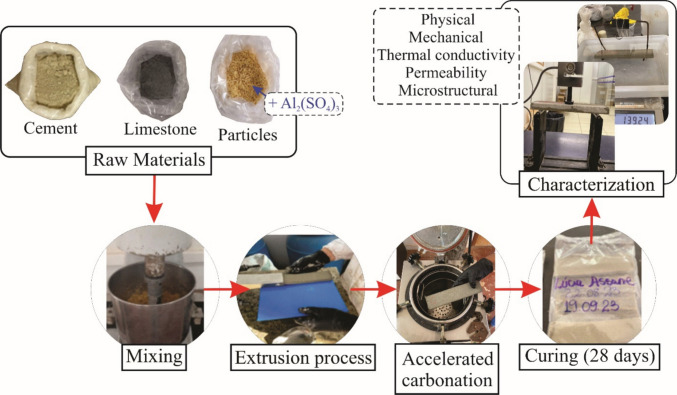
Schematic representation of the experimental procedure for the production of cement-based composites, including raw materials preparation, mixing, extrusion, accelerated carbonation, curing (28 days), and subsequent characterization (physical, mechanical, thermal, permeability, and microstructural analyses)

The materials were properly weighed and separated according to the established formulation. All components were then mixed in a planetary mixer. Initially, the cement and limestone were homogenized, followed by the addition of the lignocellulosic material, additives, and water. Homogenization was maintained for 5 min, with an angular rotation of 27.02 rad/s, aiming to ensure the uniform dispersion of particles in the cementitious matrix. The resulting mass was processed in a helical extruder from the brand VERDÉS, model 051, equipped with a screw speed controller and vacuum system, allowing for the proper shaping of the test specimens. For each treatment, six specimens (*n* = 6) were prepared for each property evaluated (physical, mechanical, and thermal), while three specimens (n = 3) were prepared for permeability and chemical analyses. Figure 1 shows the flowchart of the production process of the cementitious composites.

### Accelerated carbonation

Following production, the specimens were stored in sealed plastic bags and maintained under saturated conditions at ambient temperature (25 °C) for 72 h, a necessary step for the pre-hardening of the cementitious matrix. After this period, accelerated carbonation was performed, according to a methodology adapted from Filomeno et al. (2020). For this, an autoclave connected to an industrial cylinder of carbon dioxide (CO_2_) with 99% purity was used, via a suitable hose. During the carbonation process, a constant pressure of 0.75 MPa was maintained, with the specimens exposed to the CO₂-rich environment for 24 h. The carbonation pressure and exposure time were selected based on previous studies on accelerated carbonation of fiber-cement and cement-based materials, which report that moderate CO₂ pressures enhance CaCO₃ precipitation while maintaining matrix integrity and allowing effective gas diffusion. After this treatment, the specimens were again sealed in plastic bags and stored at ambient temperature (25 °C) for another 25 days, completing a total curing cycle of 28 days, before performing the physic-mechanical and microstructural analyses.

### Characterization of fiber cements

The physical properties of the composites, including water absorption (WA), apparent density (AD), and apparent porosity (AP), were evaluated. Specimens were initially submerged in water for 24 h to determine the immersed mass (IM) and wet mass (WM). Subsequently, they were dried in an oven at 105 °C for 24 h to determine the dry mass (DM). Calculations of the physical properties were performed according to the procedures described in ASTM C 948-81 (ASTM 1981).

Mechanical tests were performed on the specimens using a universal testing machine from Arotec, equipped with a 2 kN load cell. To determine the modulus of rupture (MOR), modulus of elasticity (MOE), and toughness, a three-point bending configuration was employed, with a fixed lower span of 132 mm. Test parameters were defined based on procedures adapted from NBR 15498 (2021).

Scanning electron microscopy (SEM) images of the fiber cement composites were obtained using a LEO EVO 40 XVP scanning electron microscope (Carl Zeiss, 2002), operating at 10 kV. Analyses were performed on the fracture surfaces of specimens subjected to static bending tests. The objective was to analyze the effects of accelerated carbonation and aluminum sulfate on the microstructural properties of the fiber cement, as well as to investigate the influence of this condition on the fiber-matrix interface.

Transmission FTIR analysis was conducted after pre-drying the powdered fiber cement samples from each treatment at 50 °C to ensure complete moisture removal. The materials were then incorporated into KBr at a 1:100 mass ratio, homogeneously mixed, and ground. FTIR spectra were acquired using a Shimadzu IRAffinity-1 spectrometer in the frequency range of 400–4000 cm⁻^1^, with a resolution of 2 cm⁻^1^. The aim of the analysis was to identify functional groups.

Thermal conductivity of the composites was evaluated following the methodology described by Batista et al. (2024). The experimental setup consisted of two superimposed chambers, both internally lined with layers of expanded polystyrene and subsequently with thermal insulation material to minimize heat exchange with the external environment and ensure uniform internal conditions. The chambers were used for controlled heating of the specimens, with continuous monitoring until thermal stabilization was achieved. During this process, temperature values were recorded on both faces of the sample: the surface exposed to lamp radiation and the opposite surface. Thermal conductivity of the composites was then determined using Eq. (1), which is based on the temperature difference and the sample’s geometry.

1 $$K=\frac{P*E}{\Delta T}$$

where *K* represents the thermal conductivity in units of W/°C; P refers to the radiation per unit area in W/m^2^; *E* represents the thickness of the test specimen in meters; and Δ*T* is the observed temperature variation in degrees Celsius.

The evaluation of air permeability was conducted using a laboratory-made air permeameter, a methodology extensively detailed in previous works (Fioroni et al. 2020). The permeability coefficients of the samples were determined by applying Forchheimer’s Eq. (2), an empirical relationship widely recognized in the literature for expressing the parabolic dependence of pressure drop through a porous medium in relation to the superficial velocity of the fluid.

2 $$\frac{\Delta P}{L}= \frac{\mu }{{k}_{1}}{ v}_{s} + \frac{\rho }{{k}_{2}} {{v}_{s}}^{2}$$

For compressible flow, the pressure drop (DP) is obtained by:

3 $$\Delta P= \frac{{{P}_{i}}^{2}-{{P}_{o}}^{2}}{{2P}_{o}}$$

where P_i_ and P_o_ are the inlet and outlet air pressures during the test, L is the sample thickness; μ indicates the fluid viscosity; ρ is the fluid density; k_1_ is the Darcian permeability coefficient, representing the material’s resistance to viscous flow, which is dominant at low fluid velocities. It is primarily influenced by the total porosity and the average pore diameter. k_2_ is the non-Darcian permeability coefficient, accounting for inertial effects that become significant at higher fluid velocities. This coefficient is more sensitive to the tortuosity and connectivity of the pore network, reflecting energy dissipation due to changes in flow direction and velocity within the complex pore structure.

Experimentally, the test involves forcing air through the sample at controlled volumetric flow rates (Q) and measuring the corresponding pressure drop (ΔP) across the sample. The collected data are then fitted to Forchheimer’s equation, allowing for the calculation of k_1_ and k_2_ from the linear and quadratic terms, respectively. This approach provides a comprehensive characterization of the material’s fluid transport properties across different flow regimes, offering a more realistic and reliable assessment compared to models that consider only viscous effects.

### Data analysis

The data obtained in this study were analyzed using a completely randomized design. Analysis of variance (ANOVA) was applied, followed by the Scott-Knott test of means, both adopting a significance level of 5% (*p* ≤ 0.05).

## Results and discussions

### Properties of lignocellulosic material

Table 2 presents the chemical composition of *Erythrina poeppigiana* particles in their natural state and after aluminum sulfate treatment, highlighting significant changes induced by the chemical process. An increase in total extractives content and a reduction in insoluble lignin were observed. This decrease in lignin contributes to the partial removal of hydrophobic components, which may enhance adhesion between fibers and the cementitious matrix. However, high levels of extractives tend to hinder interaction with cement-based materials (Silva et al. 2009).

**Table 2:** Chemical composition of *Erythrina poeppigiana* natural particles and treated with Al_2_(SO_4_)_3_

Analyses (%)	Natural particles	Treated particles Al_2_(SO_4_)_3_
Total extractives	9.54^(1.37)^	10.07^(2.04)^
Insoluble lignin	23.92^(1.03)^	12.92^(5.03)^
Cellulose	45.42^(1.53)^	56.79^(0.87)^
Hemicelluloses	19.01^(1.97)^	17.49^(3.28)^
Ash	2.11^(0.075)^	3.53^(0.078)^

Values in parentheses correspond to standard deviation

In the study by Fonseca et al. (2019), the performance of jute fibers treated with NaOH as reinforcement in extruded fiber cement composites was evaluated. The treatment reduced the lignin content to 11.07%, a value similar to that obtained in the present study using Al₂(SO₄)₃. Additionally, a significant increase in cellulose content was reported, from 53.1 to 75%, demonstrating the effectiveness of alkaline treatment in modifying the properties of natural fibers and making them more suitable for use in cementitious matrices. In this study, cellulose content increased from 45.42 to 56.79%. As the primary structural component of fibers, cellulose directly influences tensile strength and stiffness. This enrichment suggests a potential improvement in the mechanical performance of the composites (Fiore et al. 2016).

Regarding hemicelluloses, the values showed a slight decrease, from 19.01 to 17.49%, remaining relatively stable. Conversely, ash content increased from 2.11 to 3.53%, likely due to the presence of residual inorganic salts resulting from Al₂(SO₄)₃ treatment. This increase in mineral fraction may alter local alkalinity and interfere with cement hydration reactions, directly affecting the fiber-matrix interface (Ashori and Nourbakhsh 2010).

Within the context of fiber cement composites, these findings indicate that Al₂(SO₄)₃ treatment induces significant modifications in the chemical structure of the fibers. Such changes, including the maintenance of high cellulose levels and lignin reduction, can enhance compatibility with the cement matrix and may contribute to improvements in the physical and mechanical properties of the composites, particularly in terms of interfacial adhesion, durability, and overall mechanical performance.

### Properties of fiber cements

**Fig. 2:**
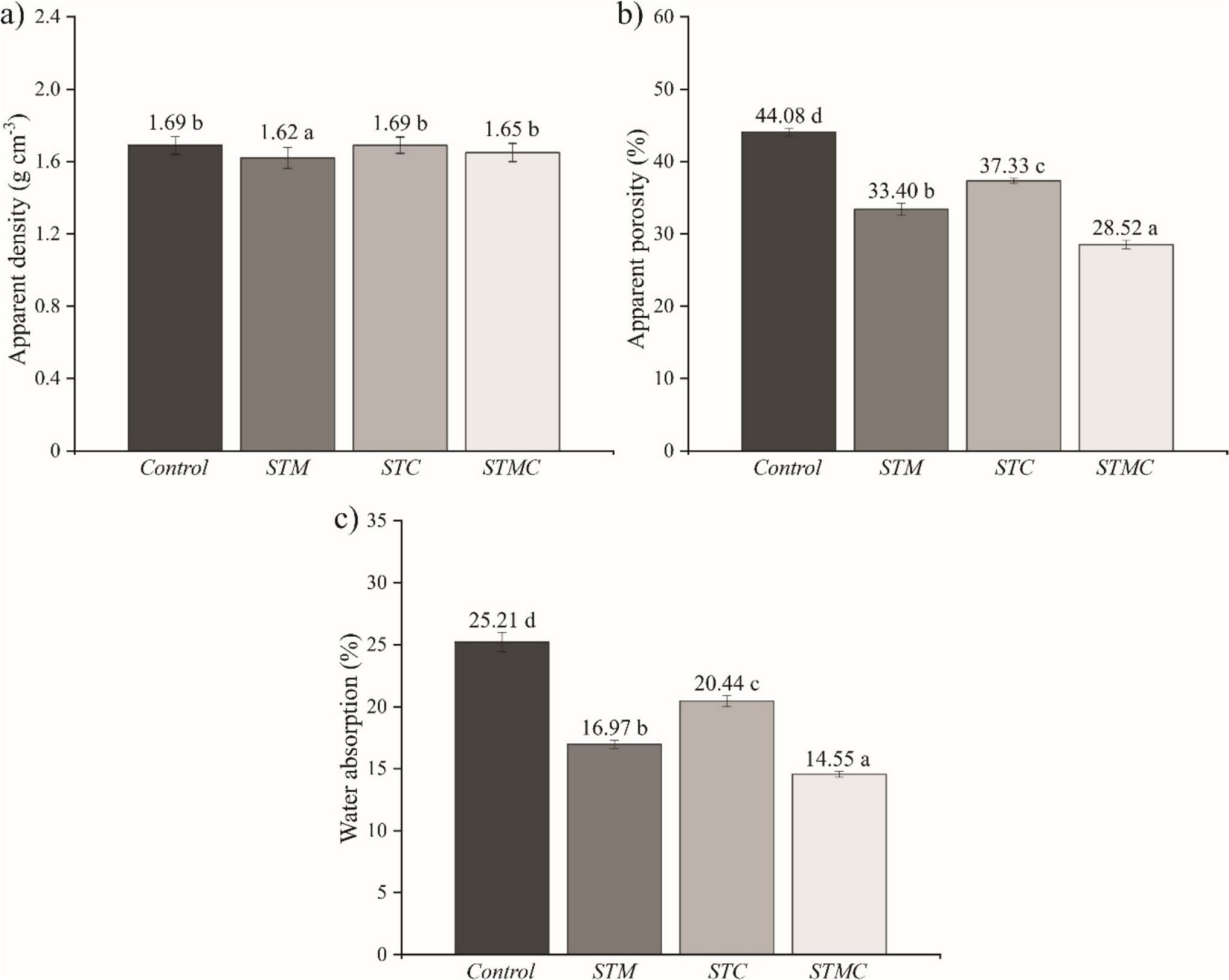
Mean values and standard deviations (error bars) of **a** apparent density, **b** apparent porosity, and **c** water absorption. Means followed by the same letter do not differ statistically according to the Scott-Knott test (*p* > 0.05)

Figure 2 presents the mean values of the physical properties of the fiber cement composites. For apparent density, the one-way analysis of variance (ANOVA) indicated a significant treatment effect (*F*_3,20_ = 7.92; p = 0.0011). The Scott-Knott test (5%) separated the treatments into two statistical groups, with STM showing lower apparent density compared to the other treatments, which did not differ from each other. Apparent density ranged from 1.62 to 1.69 g cm^−3^, indicating that the cement matrix remained relatively compact regardless of the applied treatments (Fig. 2a).

In contrast, apparent porosity showed pronounced differences among treatments (*F*_3,20_ = 656.82; *p* < 0.0001), with the highest value recorded for the control (44.08%) and the lowest for the STMC treatment (28.52%) (Fig. 2b). This reduction suggests that the combination of particles mineralized with Al₂(SO₄)₃ and accelerated carbonation contributed to a less porous structure, possibly due to a synergistic effect between the formation of reaction products and internal matrix rearrangement.

Al₂(SO₄)₃ contributes to pore volume reduction, particularly within the 5–80 nm range, owing to its accelerating effect on calcium aluminate hydration and subsequent formation of calcium monosulfoaluminate (Kan et al. 2013). This microstructural modification decreases void spaces and increases material compaction. Pantawee et al. (2017) also reported that Al₂(SO₄)₃ acts as a flocculating agent, promoting greater particle agglomeration and densification. Furthermore, the accelerated carbonation process in the present study likely reacted with hydrated compounds in the cementitious matrix, forming calcium carbonate, which further reduced porosity. When combined with carbonation, additional matrix reorganization occurs, potentially enhancing the overall properties of the composites.

Water absorption results for the fiber cement composites (Fig. 2c) exhibit a clear correlation with the previously discussed porosity data. The analysis of variance indicated a significant effect of the treatments on water absorption (*F*_3,20_ = 528.01; *p* < 0.0001). The control treatment, which had the highest porosity, also displayed the highest water absorption (25.21%). This behavior is expected, as a greater number of pores, especially interconnected ones, facilitate water penetration and retention within the material.

The lowest water absorption value was observed for the STMC treatment (14.55%), which also had the lowest porosity (28.52%). These results support the hypothesis that the combined effect of the treatments promotes pore filling with calcium carbonate in the cementitious matrix, reducing void spaces and hindering water ingress. Such structural modifications contribute to improved durability and lower susceptibility to moisture-related degradation. Similar findings have been reported in studies employing accelerated carbonation as a strategy to enhance the physicochemical properties of cement-based composites (Filomeno et al. 2020; Wang et al. 2019).

Based on the water absorption values obtained, all evaluated treatments comply with the limits established by NBR 7581-1 (2014), which sets a maximum value of 37% for fiber cement intended for the manufacture of asbestos-free roofing sheets. The values are also in line with practical performance ranges reported for commercial fiber cement products tested according to procedures referenced in ASTM C1186 (2022) and related standards, where typical water absorption for market-available fiber cement materials is generally reported at up to ~ 35%. This demonstrates the conformity of the composites with current technical standards and highlights the promising potential of the tested treatments for practical applications.

**Fig. 3:**
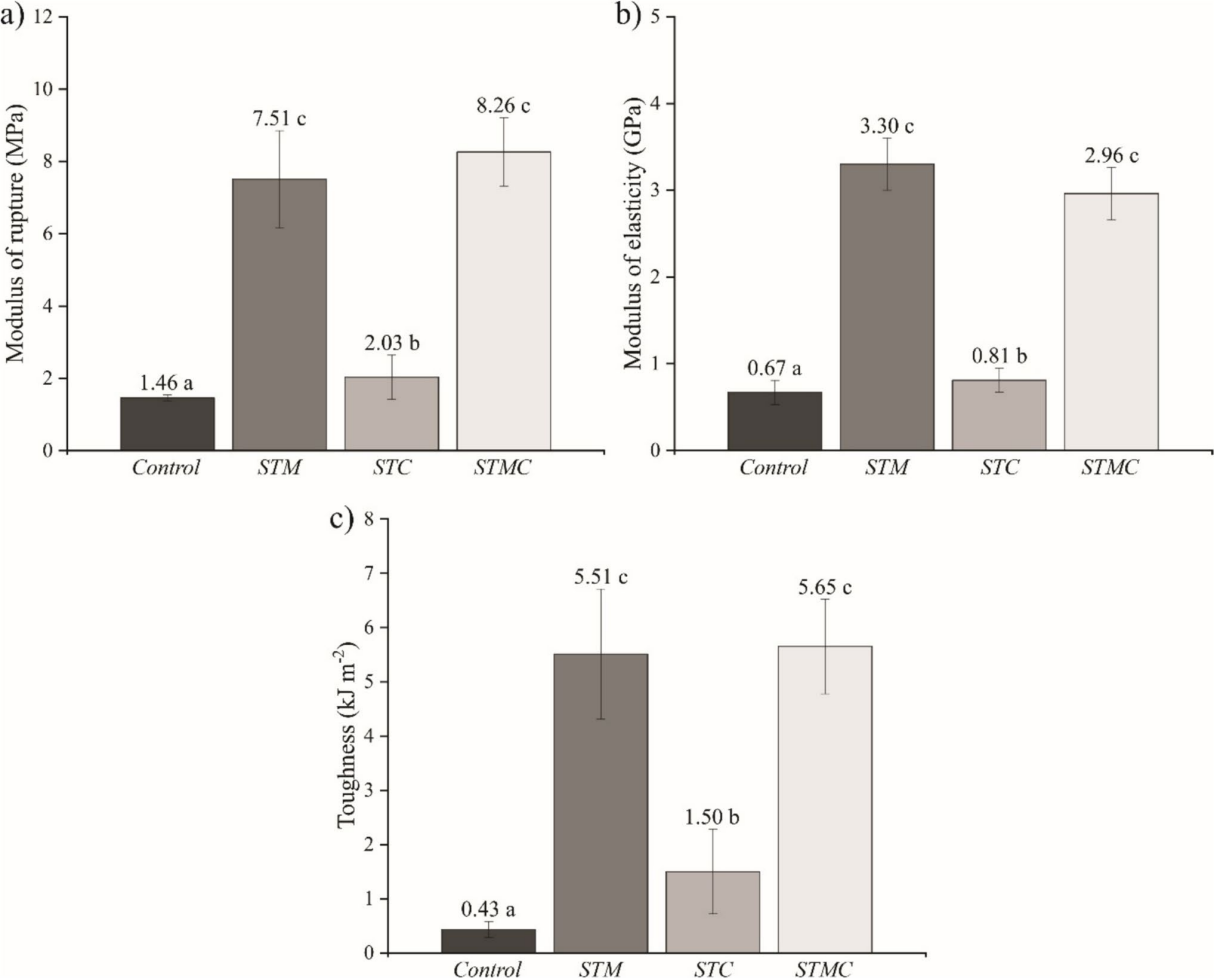
Mean values and standard deviation (error bars) of mechanical properties: **a** modulus of rupture (MOR), **b** modulus of elasticity (MOE), and **c** toughness. Means followed by the same letter do not differ statistically according to the Scott-Knott test (*p* > 0.05)

In the mechanical properties, significant differences can be observed (Fig. 3) between the treatments regarding the modulus of rupture (MOR) (*F*_3,20_ = 96.74; *p* < 0.0001), modulus of elasticity (MOE) (*F*_3,20_ = 191.48; *p* < 0.0001) and toughness (*F*_3,20_ = 56.17; *p* < 0.0001). The control treatment exhibited the lowest MOR value (1.46 MPa), reflecting its high porosity and elevated water absorption (see Fig. 2c), which result in reduced structural cohesion and, consequently, low mechanical strength. In contrast, the STM and STMC treatments promoted a significant increase in MOR (7.51 and 8.26 MPa, respectively).

Although partial densification of the matrix may have contributed to the mechanical improvement, the observed performance gains cannot be attributed solely to porosity reduction. The mineralization treatment likely promoted chemical modification of the lignocellulosic surface, improving fiber-matrix compatibility and enhancing interfacial bonding. This modification favors more efficient stress transfer between the cementitious matrix and the reinforcement phase, leading to higher flexural strength. In addition, mineral precipitation on the fiber surface may act as nucleation sites for hydration products, refining the interfacial transition zone (ITZ) and generating a more homogeneous stress distribution throughout the composite (Cui et al. 2025).

Furthermore, carbonation contributes to microstructural refinement through the formation of calcium carbonate crystals within pores and around fibers, increasing local stiffness and reinforcing the fiber-matrix interface. The reduction in alkalinity also limits the degradation of lignocellulosic components, preserving fiber integrity and enabling effective crack-bridging and pull-out mechanisms during loading. These mechanisms enhance energy dissipation capacity and contribute directly to the observed increases in MOR and toughness, beyond the effects of bulk porosity changes alone. Similar improvements associated with Al_2_(SO_4_)_3_ treatment were reported by Batista et al. (2024), who observed enhanced fiber-matrix adhesion and increased flexural strength.

The MOR values obtained for STM and STMC treatments comply with the limits established by NBR 15498 (ABNT 2021), which classifies fiber cement composites with MOR values between 7 and 13 MPa as category 3, suitable for asbestos-free board production. From an international standpoint, ASTM C1186 (2022) classifies flat fiber cement sheets into performance grades based on minimum flexural strength in the saturated condition, with thresholds of ≥ 4 MPa (grade I), ≥ 7 MPa (grade II), ≥ 10 MPa (grade III), and ≥ 13 MPa (grade IV). In this context, the MOR values obtained for the STM and STMC place both composites within grade II, which corresponds to materials suitable for general exterior use.

For MOE and toughness, the results followed the same trend, with lower values for the control and higher values for STM and STMC treatments. The flocculating effect of mineralization contributed to the formation of a structure more resistant to elastic deformation (Fig. 3b). Borges et al. (2024) reported that treatment with Al₂(SO₄)₃ in *Pinus* spp Kraft pulp increased MOE from 1.6 GPa to 2.5 GPa, corroborating the effectiveness of this modification in improving the mechanical performance of lignocellulosic composites. Similarly, Almeida et al. (2013) reported an increase in toughness in composites reinforced with plant fibers subjected to accelerated carbonation, in agreement with the present study.

The interaction between treated particles and carbonation products suggests a more homogeneous and cohesive structure that is resistant to crack propagation. The high toughness observed indicates a material with an excellent balance between strength and ductility (Fig. 3c), characteristics desirable for construction applications requiring consistent mechanical performance over time (Dai et al. 2024).

To contextualize the performance of the developed composites, Table 3 compares their key properties with those of conventional fiber cement and other bio-based cementitious composites reported in the literature.

**Table 3:** Comparison of mechanical and thermal performance of cementitious composites

Study	Material/treatment	Natural fiber	MOR (MPa)	MOE (GPa)	Toughness (kJ m—2)	Thermal conductivity (W/m·K)
This work	STMC—carbonated and mineralized	*Erythrina poeppigiana*	8.26	3.0	5.6	0.16
This work	STM—mineralized	*Erythrina poeppigiana*	7.51	3.3	5.5	0.2
Borges et al. (2024)	PKP11%AS—mineralized	Pinus Kraft pulp	8.1	2.2	2.2	NR
Gbekou et al. (2023)	M5—natural fibers	Miscanthus fibers	2.31	NR	NR	0.6
Batista et al. (2024)	SM0—mineralized	*Hevea brasiliensis*	8.7	3.8	0.8	0.1
Arvizu-Montes et al. (2025)	AFM—alfa fibers	Natural fibers	5.5	NR	NR	NR
Shah et al. (2022)	SC3—fiber content of 1.5%	Sisal	3.0	NR	NR	NR
Castillo-Lara et al. (2020)	H1.5FC—fiber content of 1.5%	Henequen plant	0.4	0.02	NR	NR
Jamshaid et al. (2022)	22—fiber content of 3%	Jute Fiber	1.2	NR	0.002	NR

*NR*, not reported

**Fig. 4:**
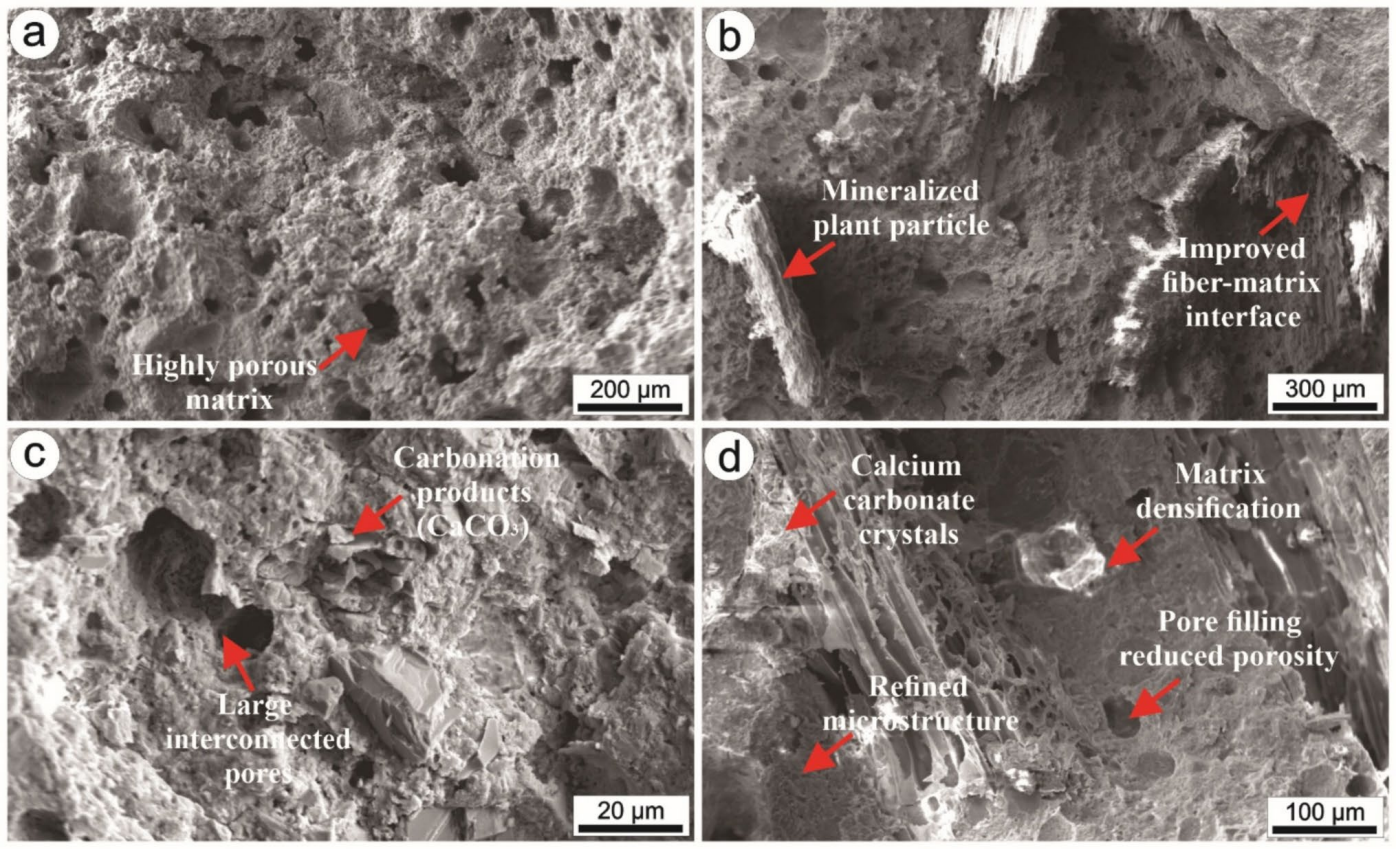
SEM micrographs of the flexural fracture surfaces of fiber cement composites. **a** Control with natural plant particles, showing a highly porous matrix; **b** STM with Al_2_(SO_4_)_3_ mineralized particles and improved fiber-matrix interface; **c** STC carbonated composite with CaCO₃ formation and pore refinement; and **d** STMC composite combining mineralization and carbonation, evidencing calcium carbonate crystals, matrix densification, and reduced porosity

Scanning Electron Microscopy (SEM) was used to characterize the microstructure of the fiber cement composites (Fig. 4). The untreated control sample (Fig. 4a) exhibited a highly porous and heterogeneous cementitious matrix, with large voids indicating limited interfacial cohesion and inefficient stress transfer between constituents. Such features are typically associated with reduced mechanical performance and higher permeability.

The STM treatment (Fig. 4b) showed mineralized plant particles and improved fiber-matrix interfacial contact compared to the control. The treatment with Al_2_(SO_4_)_3_ appears to have modified the particle surfaces, promoting better adhesion with the cementitious matrix and resulting in more continuous interfacial bonding. Although porous regions are still present, the microstructure indicates enhanced matrix-particle interaction and improved interfacial cohesion, which may contribute to more efficient stress transfer between the reinforcing particles and the cement matrix.

For the STC treatment (carbonation alone) (Fig. 4c), the formation of carbonation products (CaCO₃) was observed, along with localized pore refinement. Despite these microstructural changes, interconnected pores are still visible, indicating that carbonation alone did not fully eliminate porosity. In contrast, the STMC composite (Fig. 4d) exhibited the most refined microstructure, characterized by calcium carbonate crystal formation, matrix densification, pore filling with reduced porosity, and improved interfacial continuity. These features indicate that the combined mineralization and carbonation treatments promoted a more compact and homogeneous structure.

**Fig. 5:**
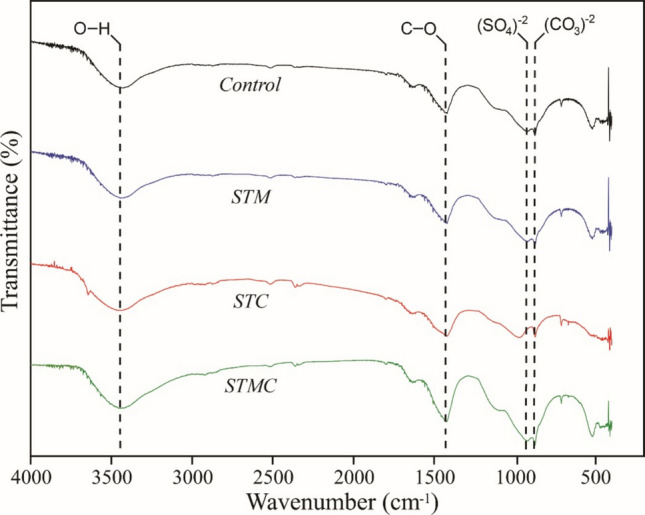
Fourier transform infrared spectroscopy (FTIR) analysis of fiber cements

Using infrared spectroscopy, it was possible to verify the presence of functional groups in the treatments performed (Fig. 5). The bands between ~3441–3446 cm⁻^1^ are associated with O-H stretching vibrations, related to absorbed water or hydroxyl groups in calcium hydroxide (portlandite) (Chen et al. 2021). The reduced intensity in the treated samples (particularly STMC) indicates a decrease in free hydroxides, suggesting carbonation of portlandite and subsequent formation of calcium carbonate (CaCO₃). SEM images of the STMC composite (Fig. 4d) revealed the presence of well-defined crystalline deposits within voids and along the fiber-matrix interface, which are consistent with CaCO₃ precipitation.

The region around ~ 1420–1470 cm⁻^1^ corresponds to the asymmetric stretching of carbonate groups (CO₃^2^⁻) (Dias et al. 2024). The increased intensity of this band in treated samples confirms the formation of CaCO₃ (notably in the SEM micrographs of STC and STMC), indicating that the composites underwent carbonation, a desirable phenomenon for enhancing durability, as it reduces matrix alkalinity and protects lignocellulosic material.

The band in the ~ 1100–1000 cm⁻^1^ range is associated with sulfate groups (SO₄^2^⁻), likely originating from aluminum sulfate (Al₂(SO₄)₃) used in treatments (Borges et al. 2024). This band is more evident in STM and STMC, suggesting interactions with sulfate salts, which may influence the matrix structure and the interface with *Erythrina poeppigiana* particles.

The bands at ~ 870 and 710 cm⁻^1^ (CO₃^2^⁻) are attributed to out-of-plane bending vibrations of carbonate groups (Zhang et al. 2021). The greater intensity observed in these regions, especially in STC and STMC, reinforces the evidence of more pronounced carbonation.

Furthermore, the filling of microvoids, coupled with the formation of calcium carbonate (CaCO₃) and the refinement of the pore structure, significantly improves the interfacial bonding between mineralized particles and the cement matrix. This densification of the microstructure enhances stress-transfer efficiency under bending loads, providing a mechanistic basis for the simultaneous increases in modulus of rupture (MOR), modulus of elasticity (MOE), and toughness observed in the composites subjected to the combined treatment. These effects demonstrate a clear synergistic interaction between aluminum sulfate mineralization and accelerated carbonation, highlighting how chemical stabilization of the fibers and matrix densification jointly contribute to superior mechanical performance.

**Fig. 6:**
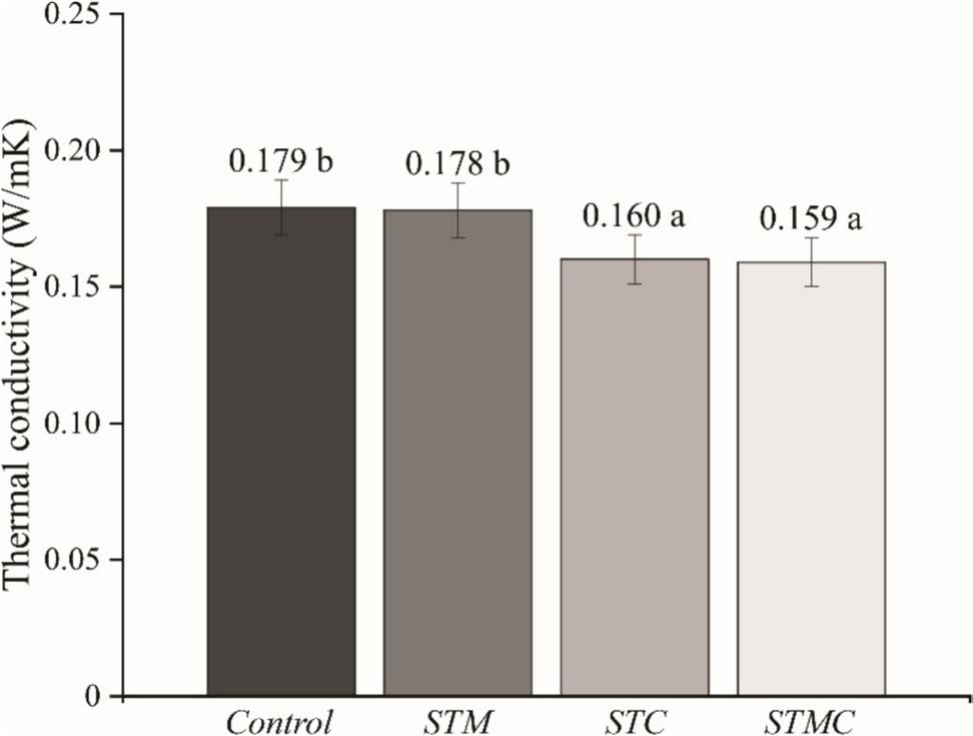
Mean values and standard deviation (error bars) of thermal conductivity of fiber cement. Means followed by the same letter do not differ statistically according to the Scott-Knott test (*p* > 0.05)

The thermal conductivity of the fiber cement composites varied significantly (*F*_3,20_ = 77.53; *p* < 0.0001) as a function of the treatments applied to the lignocellulosic particles and the cementitious matrix (Fig. 6). Composites subjected to accelerated carbonation, both with untreated particles (STC) and with previously mineralized particles (STMC), exhibited significantly lower thermal conductivity values compared to the control and STM (without carbonation), with mean values of 0.160 and 0.159 W/m·K, respectively. These results indicate that carbonation plays a decisive role in modifying the composite microstructure, inducing changes that directly impact the material’s heat conduction capacity.

The reduction in thermal conductivity in carbonated composites may be associated with the formation of calcium carbonate (CaCO₃) within the matrix pores. This carbonation process tends to consume calcium hydroxide, altering the mineralogical composition of the matrix and refining its microstructure, which may contribute to decreased thermal diffusivity (Smith et al. 2013).

Eugenio et al. (2023) reported thermal conductivity values ranging from 0.48 to 0.52 W/m·K in extruded concrete tiles incorporating mining waste. These values are considerably higher than those obtained in the present study, indicating superior thermal performance for the developed composites. Low thermal conductivity, and consequently a greater temperature gradient across the interface between the cementitious matrix and plant particles, is a desirable characteristic in the construction sector. This limitation in heat transfer between the interior and exterior faces of buildings contributes to improved indoor thermal comfort and reduced energy consumption associated with heating or cooling (Viana et al. 2022).

Conversely, the STM treatment, which involved particles mineralized with Al₂(SO₄)₃ without subsequent carbonation, did not exhibit a statistically significant difference compared to the control (0.178 and 0.179 W/m·K, respectively), suggesting that mineralization alone does not substantially affect thermal conductivity. This indicates that the introduction of the mineralizing agent, although beneficial for other properties such as durability and matrix adhesion, does not significantly alter heat conduction mechanisms within the composite.

Overall, the findings demonstrate that carbonation is the primary factor responsible for reducing the thermal conductivity of the studied composites, highlighting its potential for application in construction systems requiring enhanced thermal insulation. This property, combined with other benefits of carbonation, such as improved dimensional stability and durability, reinforces the feasibility of carbonated composites as sustainable and functionally improved alternatives to conventional materials.

**Fig. 7:**
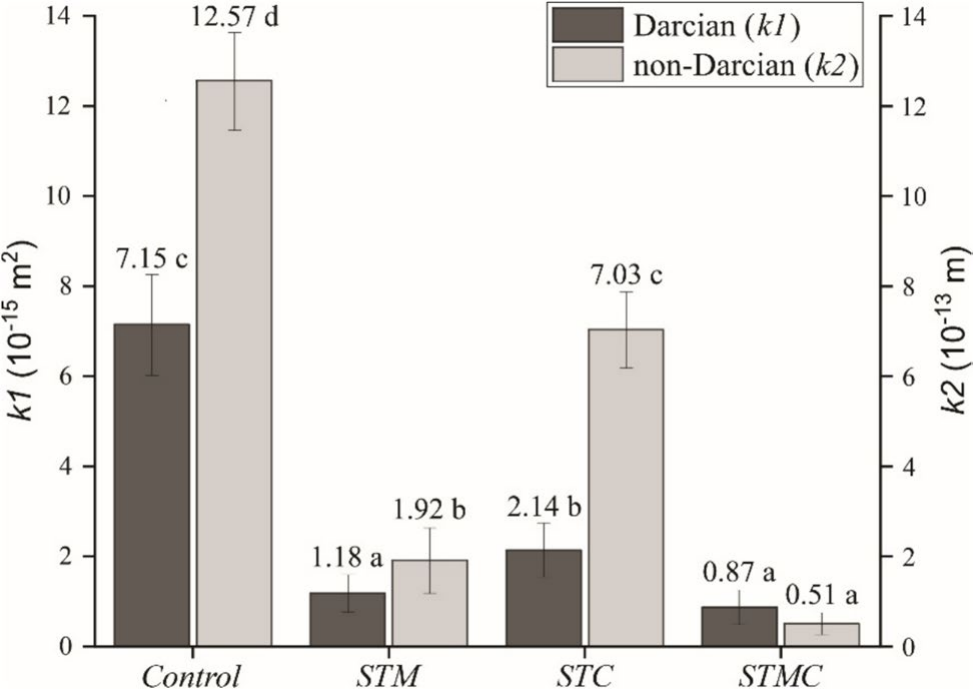
Mean values and standard deviation (error bars) of fiber cement permeability. Means followed by the same letter do not differ statistically according to the Scott–Knott test (*p* > 0.05)

The permeability analysis of the fiber cement composites, conducted using the established Forchheimer’s equation methodology (Innocentini et al. 2022), revealed significant differences among the treatments in both the linear (Darcian, *k*_1_) (*F*_3,80_ = 60.19; *p* < 0.0001) and non-linear (non-Darcian, *k*_2_) (*F*_3,80_ = 64.91; *p* < 0.0001) flow regimes. The control treatment exhibited the highest permeability values, reflecting a more porous matrix with greater pore connectivity, which facilitates fluid flow. In contrast, the modified treatments showed substantial reductions in these parameters (Fig. 7).

The STM treatment, which employed particles mineralized with Al₂(SO₄)₃, resulted in a marked decrease in permeability, particularly for the *k*₁ coefficient (1.18 × 10⁻^15^ m^2^), indicating that the mineralization process promotes pore obstruction or matrix filling by secondary phases formed during hydration. Similarly, the STC treatment, based on untreated particles subjected to carbonation, also reduced permeability compared to the control, likely due to the formation of carbonates that partially block the matrix pore network.

The STMC treatment, which combined particle mineralization with carbonation, achieved the lowest permeability values (*k*₁ = 0.87 × 10⁻^15^ m^2^; *k*₂ = 0.51 × 10⁻^13^ m), demonstrating a synergistic effect between the two processes in controlling effective porosity. These findings align with those reported by Fioroni et al. (2020), who observed reduced permeability coefficients following accelerated carbonation in corrugated fiber cement sheets.

This substantial reduction indicates a denser and less permeable microstructure, which can directly enhance the durability of the composites against aggressive agents, consistent with the apparent porosity and water absorption results (see Fig. 2). Therefore, these findings reinforce the potential of combining carbonation with mineralization as an effective strategy to improve the performance of cementitious composites, particularly in applications requiring low permeability and increased resistance to environmental degradation.

**Fig. 8:**
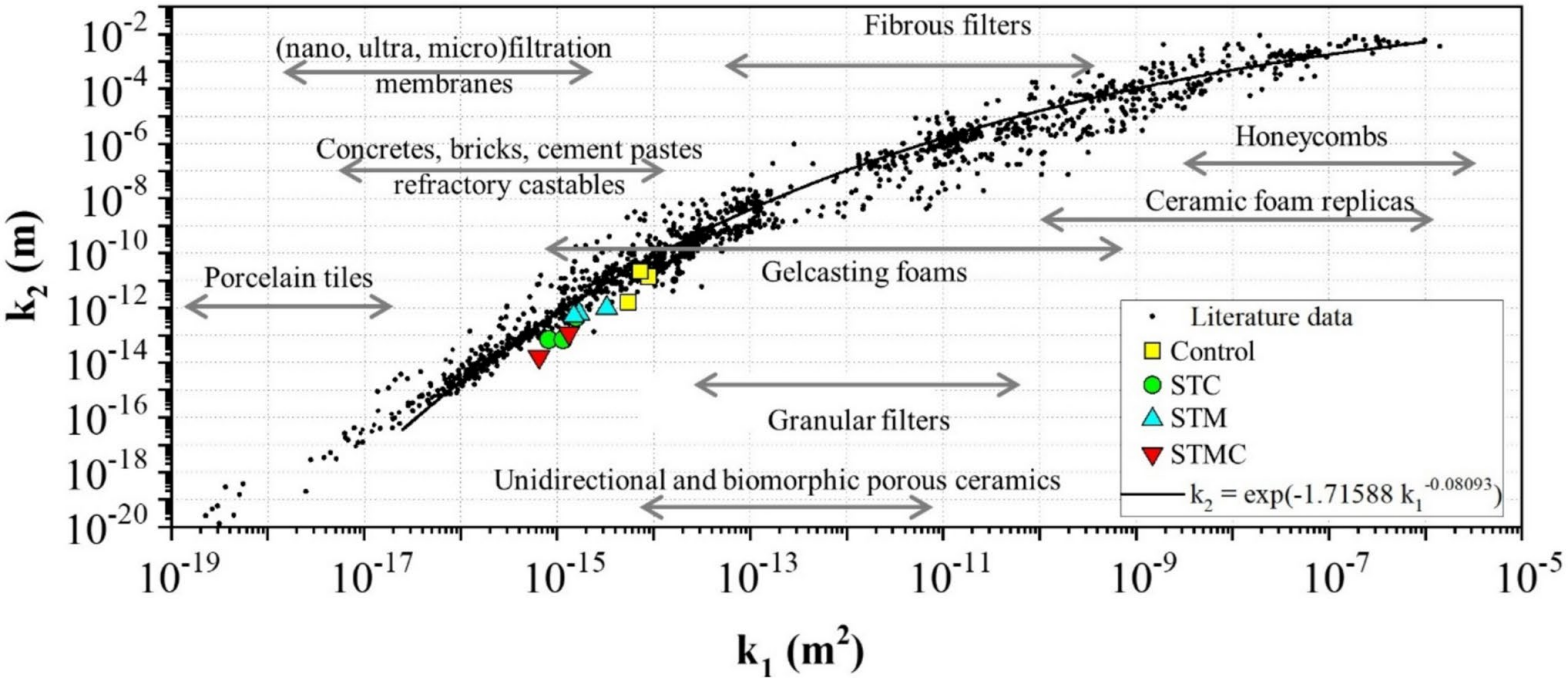
Permeability map classifying various porous materials and highlighting the position of the fiber cement composites evaluated in this study

Figure 8 presents a comprehensive permeability map, compiling over 2000 datasets from diverse porous materials to illustrate the relationship between Darcian (*k*₁) and non-Darcian (*k*₂) permeability coefficients. Our fiber cement composites, particularly the STMC treatment, exhibit *k*₁ values of 0.87 × 10⁻^15^ m^2^ and *k*₂ values of 0.51 × 10⁻^13^ m. These results consistently place them within the low permeability range characteristic of dense cement-based materials like concrete, mortars, bricks, and refractories.

The general fitting curve (*k*₂ = exp(−1.71588 *k*₁⁻⁰·⁰⁸⁰⁹^3^)) on this map represents a broad empirical correlation across a vast and heterogeneous dataset. The observed position of our composites, which appears below this general trend, is a direct consequence of the successful microstructural densification and pore network refinement achieved through the combined mineralization and accelerated carbonation treatments. As supported by our apparent porosity, water absorption, and microstructural analyses (see Figs. 2, 4, and 5), these treatments effectively reduce void spaces and enhance matrix cohesion. This outcome underscores the efficacy of our approach in developing high-performance composites with superior resistance to fluid ingress, aligning with the expected behavior of optimized, low-permeability cementitious materials.

However, some limitations should be considered. Although the results obtained in this study indicate significant improvements in properties commonly associated with durability, such as reduced porosity, lower water absorption, decreased permeability, and enhanced microstructural densification, the long-term durability of the composites under real environmental conditions was not directly evaluated.

In particular, critical durability aspects for fiber cement materials, including resistance to wet–dry cycles, alkali degradation of lignocellulosic components, and long-term aging, were not experimentally assessed in this study. Nevertheless, the substantial reduction in pore connectivity and permeability, combined with the formation of calcium carbonate and the improved fiber-matrix interface observed in SEM analyses, suggest a strong potential for enhanced durability performance.

Previous studies have demonstrated that carbonation reduces matrix alkalinity and limits fiber degradation, while mineralization treatments contribute to the stabilization of lignocellulosic materials in cementitious environments. Therefore, the synergistic effects observed in the present study are expected to positively influence long-term durability, although this assumption requires direct experimental validation.

Future research should focus on evaluating the durability of these composites under aggressive environmental conditions, including cyclic wetting and drying, thermal variations, and long-term exposure to alkaline environments, as well as assessing their long-term mechanical stability. Finally, future studies should quantify the overall environmental implications of the treatment, particularly the net CO₂ balance associated with accelerated carbonation, and conduct broader life-cycle assessments to better define the sustainability potential of the proposed material.

## Conclusion

The results demonstrate that mineralization of *Erythrina poeppigiana* particles with Al_2_(SO_4_)_3_ induces relevant chemical modifications, including reduced lignin content and increased cellulose exposure, which improve compatibility with the cement matrix and enhance fiber-matrix adhesion. When combined with accelerated carbonation (STMC), this treatment promotes microstructural densification through calcium carbonate formation, as supported by SEM and FTIR analyses.

This synergistic effect resulted in substantial improvements in composite performance. The STMC treatment significantly increased modulus of rupture, modulus of elasticity, and toughness, while simultaneously reducing apparent porosity, water absorption, thermal conductivity, and air permeability, thereby significantly enhancing resistance to fluid ingress and degradation. These changes indicate a denser and more cohesive matrix, improved stress transfer at the interface, and enhanced resistance to fluid ingress, contributing to both mechanical reliability and durability-related properties. The reduced thermal conductivity further suggests potential benefits for building envelope applications requiring improved thermal performance.

## Data Availability

The data supporting the findings of this study are available upon request to the corresponding author(s).
